# Light-dependent switching between two flagellar beating states selects versatile phototaxis strategies in microswimmers

**DOI:** 10.1073/pnas.2408082121

**Published:** 2024-11-13

**Authors:** Alan C. H. Tsang, Ingmar H. Riedel-Kruse

**Affiliations:** ^a^Department of Mechanical Engineering, The University of Hong Kong, Hong Kong, China; ^b^Department of Molecular and Cellular Biology, University of Arizona, Tucson, AZ 85721; ^c^Department of Applied Mathematics, University of Arizona, Tucson, AZ 85721; ^d^Department of Physics, University of Arizona, Tucson, AZ 85721; ^e^Department of Biomedical Engineering, University of Arizona, Tucson, AZ 85721

**Keywords:** phototaxis, microswimmer, flagellar beating, adaptation, *Euglena*

## Abstract

Microorganisms have evolved sophisticated sensor-actuator circuits to perform taxis in response to various environmental stimuli. How any given circuit can select between different taxis responses in noisy vs. saturated stimuli conditions is unclear. Here, we investigate how *Euglena gracilis* can select between positive vs. negative phototaxis under low vs. high light intensities, respectively. We propose three general selection mechanisms for phototactic microswimmers, and biophysical modeling demonstrates their effectiveness. Perturbation and high-speed imaging experiments show that of these three mechanisms, the “photoresponse inversion mechanism” is implemented in *E. gracilis*: a fast, light-intensity-dependent switching between two flagellar beat states responsible for swimming and turning causes positive vs. negative phototaxis at low vs. high light intensity via run-and-tumble vs. helical klinotaxis strategies, respectively. This coordinated beat-switching mechanism then also accounts for a larger set of previously reported *E. gracilis* behaviors; furthermore, it suggests key design principles for other natural as well as synthetic microswimmers.

## Significance

Microswimmers are able to generate a large set of complex behaviors in response to external stimuli in order to navigate their environment. How these different behaviors can be achieved within a single cell by a rather simple sensor-actuator circuit is poorly understood. Here, we combine experiments and modeling to study this question for *Euglena gracilis*, a single-cell organism that responds to weak and strong light to perform positive and negative phototaxis, respectively. We find that a simple “photoresponse inversion mechanism” initiates a temporal sequence of switching between just two flagellar beat states (for swimming vs. turning) and thereby generates phototaxis and many other complex behaviors. This work informs behavior selection in other species, with significant implications for ecology and sustainability.

Uni- and multicellular microswimmers play a key role in our ecosystems, furthermore have many applications for supporting bioremediation and a sustainable bioeconomy (1–5). These microswimmers navigate their environment, and they have evolved versatile feedback control strategies that allow them to select from various motility behaviors in response to environmental stimuli (6–24). For example, bacteria alternate between running and tumbling states to navigate chemical gradients (12–16), *Chlamydomonas reinhardtii* coordinate their *cis* and *trans* flagellum to transition between positive and negative phototaxis (17–22), and *Volvox carteri* control their positive and negative phototaxis by accelerating or ceasing their anterior flagella (23, 24). A variety of organisms of different body sizes and flagella numbers are currently studied, promising the understanding of generalizable biophysical laws as well as a versatile solution spectrum to “navigation problems” (25, 26). How these various microswimmers ultimately can select between different motility behaviors in a stimulus-dependent manner, e.g., switching between positive and negative taxis, is not well understood.

**Fig. 1.**
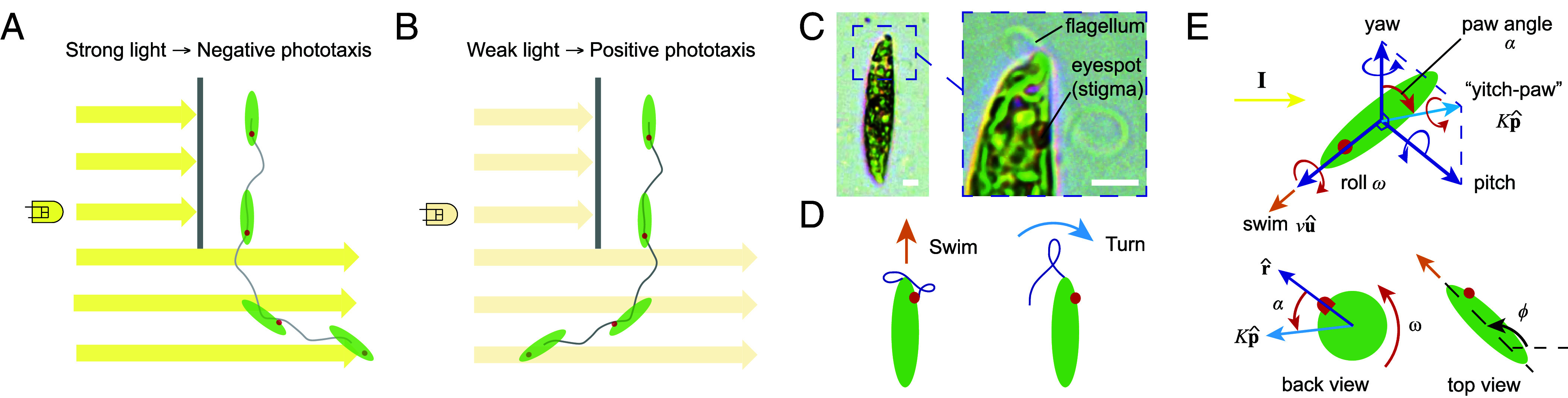
How microswimmers like *E. gracilis* achieve the selection between negative and positive phototaxis for different light intensities is unclear. (*A*) Schematic of *E. gracilis* performing negative phototaxis to swim away from strong light intensity. (*B*) Schematic of *E. gracilis* performing positive phototaxis to swim toward weak light intensity. (*C*) *E. gracilis* has a flagellum responsible for motility and red eyespot (stigma) associated with a photoreceptor. (Scale bars, 5 μm.) (*D*) *E. gracilis* has two main beat patterns that primarily achieve forward swimming and sideways turning behavior (11). (*E*) Schematic of key vectors and parameters to mathematically describe any biophysical feedback model between photosensor and flagellum in relationship to cellular position, orientation, and movement in lab reference frame (see for additional details in ref. 11): $\hat{r}$, $\hat{u}$, $\hat{p}$: orientation of cell; I: direction and intensity of light source (parallel to 2D lab plane if not stated otherwise); photoreceptor senses I, and where $\hat{r}$ denotes the receptor’s direction of maximal sensitivity; signal is converted into a specific flagellar beat pattern (*D*), which affects swimming speed v (“surge”) along the long axis $\hat{u}$, rolling frequency ω (“roll,” positive for anticlockwise rotation) around $\hat{u}$, and side-way turning (“yitch-paw”) around $\hat{p}$ with strength K via modulation of a paw angle α in a light-dependent manner. ϕ is the projection angle of cell orientation in the 2D plane of lab frame. See *SI Appendix* for model parameters and details.

*Euglena gracilis* is an important model to study these questions (4, 5, 8–11, 27). This single-celled organism can perform positive and negative phototaxis at very low and high light intensities, respectively (i.e., from <50 lx to >10,000 lx) (Fig. 1 *A* and *B*) (8–10, 28). *E. gracilis* relies on this transition to optimize photosynthesis, to avoid photodamage by strong light, and to regulate signal transductions that stimulate biological processes (8, 10, 29–32). *E. gracilis* has an ellipsoidal shape with length and diameter of ∼50 μm and ∼10 μm, respectively, and has one or multiple photosensors situated at or close to the “eyespot” that then signal to the main flagellum to coordinate propulsion and steering; the flagellum beats with 20 to 40 Hz (10, 33, 34). *E. gracilis* swims in direction of its long-axis at ∼50μm/s while rolling around this long axis at a frequency of ω≈1 Hz (11, 35, 36) (Fig. 1*C*). *E. gracilis* is a “puller” (17), i.e., the flagellum is situated at the front of the cell from the perspective of the swimming direction (Fig. 1*C*), allowing the cell to be pulled forward or to be turned sideways. We recently detailed two distinct, photoresponsive flagellar beating states for *E. gracilis* that are responsible for forward swimming and sideways turning, respectively (Fig. 1*D*) (11); aspects of both states had been reported before but in far less detail (27, 37–40). Multiple photoreceptors [e.g., flavins, photoactivated adenylyl cyclase (PAC)] and various signaling pathway components have been identified, yet many questions regarding these sensors remain (10, 41, 42).

A significant number of different photoresponsive behaviors have been reported for *E. gracilis*, e.g., positive and negative phototaxis (Fig. 1 *A* and *B*) (8, 10, 29, 30), polygonal swimming motion (11), localized spinning (11), ordered motion in polarized light (43), avoidance turning when encountering a light barrier (11, 35, 36); furthermore, a “step-down response” vs. “step-up response” to a decrease vs. an increase in light intensity at a low vs. high background light intensity, respectively, and which typically relaxes back to swimming after stimulus reversal or a longer adaptation time (10, 27, 44). Multiple of these behaviors have been suggested to be directly caused by the coordinated switching between just two beating states (11) as well as two step-responses (8), and where cellular structures like the stigma or the green organelles (Fig. 1*C*) partially shade these photosensors depending on the light direction relative to the cell body orientation, ultimately leading to the desired taxis (Fig. 1*E*) (8, 45). In contrast, some authors claim experimental evidence that questions this mechanism (27, 46), for example, given how cells respond to polarized light or multiple simultaneous light sources (47–49), or since allegedly no “directional positive phototaxis” was ever observed (and which is considered to be distinct from “photoaccumulation”) (50). Various biophysical models have been proposed to simulate and test potential mechanisms (e.g., refs. 11, 45, and 51); any such model needs to mathematically connect the cellular orientation to the light stimulus hitting the sensor and its actuating effect on cell orientation and movement (Fig. 1*E*) (11). Overall, a coherent mechanism underlying this sensor-actuator feedback to generate these various photobehaviors under various light stimulus conditions is still lacking—both from a molecular signaling as well as from a general information processing perspective.

In this paper, we now focus on the long-standing questions on how *E. gracilis* achieves positive vs. negative phototaxis at low vs. high light intensities, respectively (Fig. 1 *A* and *B*), and how a cell can select between both behaviors depending on overall light conditions (8, 10). We take a fresh approach by using high-speed imaging and biophysical modeling. We propose three general mechanisms capturing phototaxis transition in microswimmers and verify the relevant mechanism(s) for *E. gracilis* by comparing our theoretical results with our experiments. We investigate flagellar beat responses upon step-ups or step-downs in light intensity at different overall light intensity levels, and how the cell then can achieve a set of different phototaxis strategies. We conclude that in *E. gracilis* a single mechanism for the coordinated switching between two flagellar beat states explains a large number of short- and long-term photobehaviors as well as search strategies in response to a wide range of light intensities; furthermore, positive vs. negative phototaxis are achieved via a biased random walk vs. a directional helical klinotaxis, respectively (52, 53).

## Results

### Negative vs. Positive Phototaxis.

**Fig. 2.**
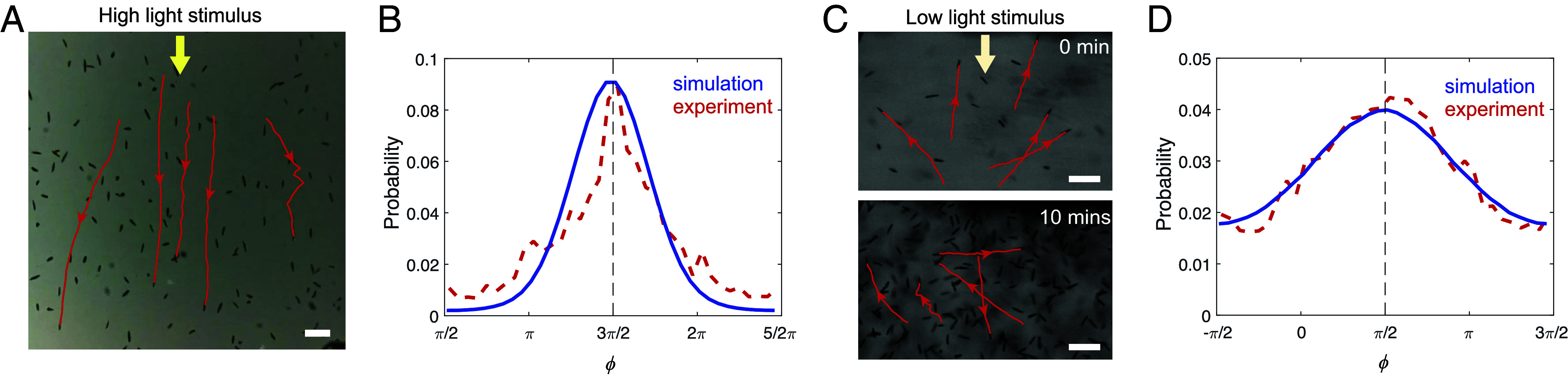
*E. gracilis* exhibits directional negative phototaxis and noisy positive phototaxis. (*A*) During negative phototaxis, the cells exhibit directed swimming away from a source of high light intensity (Movie S1). (*B*) Cell orientation ϕ during negative phototaxis shows directed swimming away from light, with a sharp peak in the light direction. (*C*) During positive phototaxis, the cells do not exhibit directed swimming toward the light source; instead, they slowly accumulate close to the light source over ∼10 min (Movie S2). (*D*) Cell orientations during positive phototaxis show a wide distribution, with a weak bias toward the light. The red lines in (*B*) and (*D*) depict the experimental results obtained from the tracking data in a 1 and 5 min interval, respectively, with each cell tracked over 3 s. 406 and 1,320 cells were tracked in experiments in (*B*) and (*D*), respectively. The blue lines in (*B*) and (*D*) depict the results obtained from Monte-Carlo type simulations of 1,000 runs with duration of 600 time units. See *SI Appendix*, Texts 3.9 and 4.2 for model parameters and additional details. [Scale bars, (*A*) 100 μm and (*C*) 100 μm.].

First, we experimentally recorded *E. gracilis*’s swimming trajectories and population responses during positive and negative phototaxis (Fig. 2). White light stimuli were applied from the side of the observation chamber with background light from below (see *SI Appendix* for full experimental methods). Negative phototaxis swimming trajectories were parallel to the light vector with slight oscillations due to cellular rotation (Fig. 2 *A* and *B* and Movie S1; background ∼100 lx, stimulus >2,000 lx). Positive phototaxis trajectories appeared almost random with frequent turns of ∼5 to 10 s and a weak bias in orientation of ϕ∼π/2 toward the light [Fig. 2 *C* and *D* and Movie S2; background ∼0 lx, stimulus <50 lx; cells were imaged under red light which does not cause responses (28)]. Over the time scales of 5 min the cells accumulated close to the light source (Fig. 2*C* and Movie S2). The orientation distributions for negative phototaxis was much narrower than that for positive phototaxis, i.e., $\overset{¯}{\phi }=4.62\pm 0.04$ (mean ± SEM, throughout the paper if not stated otherwise) vs. $\overset{¯}{\phi }=2.69\pm 0.09$, P<0.0001 (Fig. 2 *B* and *D*). At medium light intensities (∼100 lx), cells showed no phototaxis. Previously, we also reported on polygonal swimming (∼500 to 1,500 lx) and localized spinning (>3,000 lx)—both without observing any phototaxis (11). The resulting statistical distributions are consistent with earlier reports (Fig. 2 *B* and *D*) (9, 10, 30). The trajectories of individual *E. gracilis* cells and their flagellar beat patterns can also be tracked with high-speed imaging and higher magnification (Movie S3). We conclude that positive vs. negative phototaxis appear to be achieved via a biased random walk vs. directional steering (helical klinotaxis), respectively.

### Modeling Possible Phototaxis Selection Mechanisms.

**Fig. 3.**
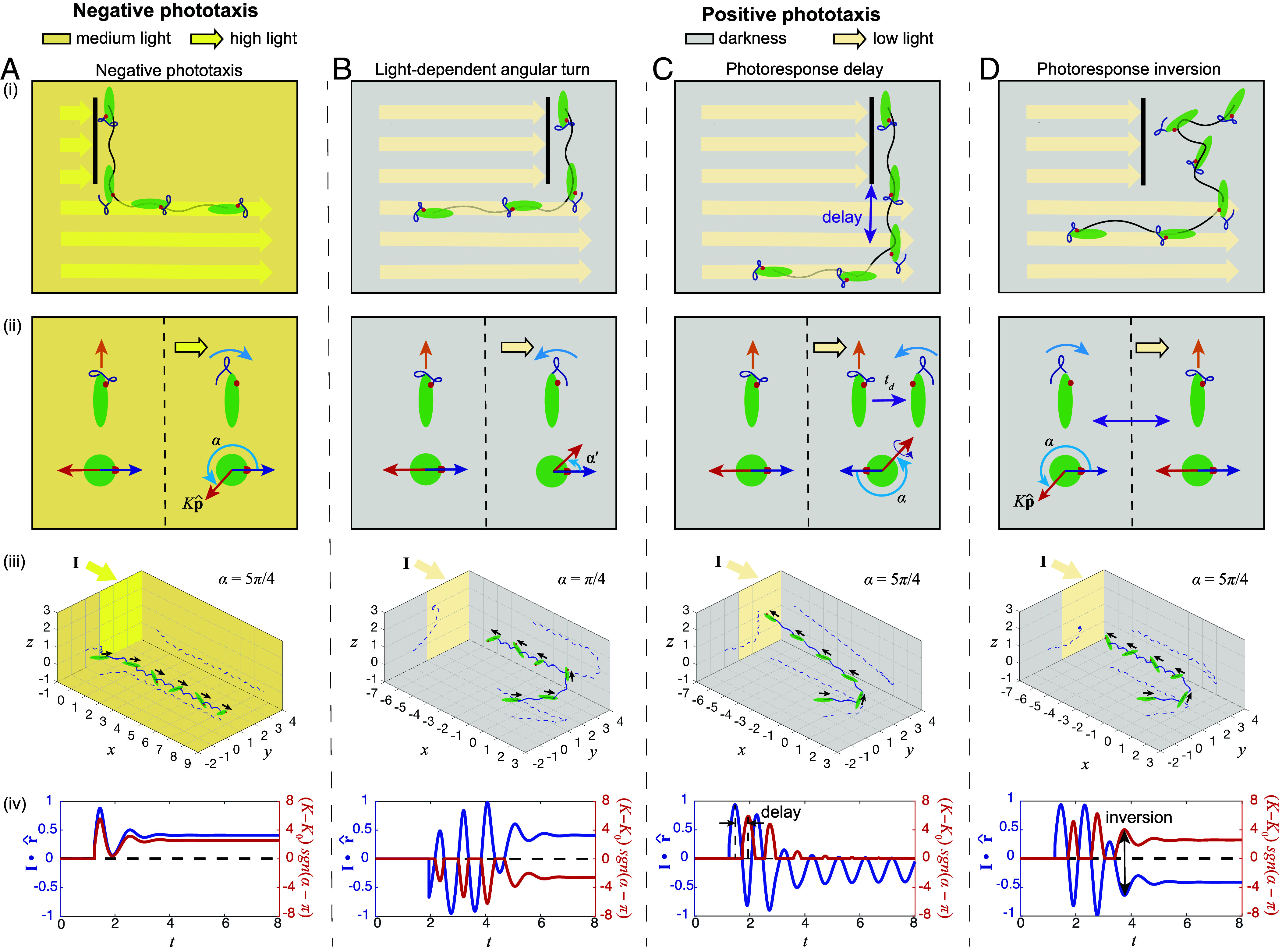
We propose and model three general mechanisms that could explain the transition between negative phototaxis and positive phototaxis. (*A*) Negative phototaxis is due to the tuning of paw angle α (Fig. 1*E*), where *E. gracilis* switches its beat pattern from forward swimming beats to sideways turning beats whenever it detects a strong light signal, leading to corresponding course corrections. (*B*–*D*) Positive phototaxis is hypothesized to be due to one of three possible general mechanisms: (*B*) Light-dependent angular turn suggests that *E. gracilis* tunes its paw angle α to a different direction as negative phototaxis and modulates its helical paths to achieve positive phototaxis. (*C*) Photoresponse delay suggests that *E. gracilis* achieves positive phototaxis by a delay mechanism in response to a light stimulus. (*D*) Photoresponse inversion suggests that *E. gracilis* inverts its response toward high light and low light, resulting in an opposite alternation of swimming and turning states between positive phototaxis and negative phototaxis. The panels in i) illustrate typical swimming paths. The panels in ii) illustrate the response of the flagellar beat patterns at different light intensities. The panels in iii) show the simulation results. Initially, each cell swims into a directional light field I as indicated by the corresponding colored region in the z-plane; trajectories are projected onto the three planes with dashed lines. The panels in iv) depict the light level as detected by the light sensor ($I\cdot \hat{r}$) as well as the turning response $\left(K-K_{0}\right)sgn\left(\alpha -\pi \right)$ that represents the light-dependent reorientation rate and thereby the direction of cell turning. See *SI Appendix*, Text 3.9 for details of model parameters.

These data raise the question of what feature in the sensor-actuator circuit (Fig. 1*E*) changes when performing positive instead of negative phototaxis, i.e., operating in a very low instead of high light intensity environment, and we propose three possible “selection mechanisms” (Fig. 3*B*–*D*): 1) Light-dependent angular turn: the paw angle α, which determines the orientation of the yitch-paw vector around which the cell turns upon detection of light (Fig. 3*A*), is changing, for example, it increases by π from α to $\alpha^{\prime }$ (Fig. 3*B*). 2) Photoresponse delay: the cell’s turning response upon detecting light is delayed, e.g., by half a roll cycle (Fig. 3*C*). 3) Photoresponse inversion: the effect of light stimuli onto the flagellar beating states for swimming and turning (Fig. 1*D*) are swapped, i.e., the cell frequently turns in the dark (step-down response) but swims straight when it detects light (step-up response) (Fig. 3*D*). Hence for all three mechanisms, the cell should be able to turn toward the light source instead of away from it, therefore leading to positive phototaxis at low light intensities.

To further substantiate the feasibility of these three possible mechanisms, we extended our previous biophysical model (Fig. 1*E*) (11) to also execute each of these mechanisms for positive phototaxis at low light intensities (Fig. 3*B*–*D* and *SI Appendix*, Texts 3.1–3.6). In our original model, the cell reorients with increased turning rate K (Fig. 1*E*) based on the detected light signal $I\cdot \hat{r}$ (light direction and intensity I and the photosensor pointing at $\hat{r}$). The model is nondimensionalized in space and time based on the body length ℓ and the rolling period 2π/ω, with v held constant. The coupling constants $K_{d}$ are tuned such that the magnitude of $K_{d}\left|I\right|$ approximately equals the light intensity in lx. If α is confined to −ϵ/2<α<ϵ/2 and π−ϵ/2<α<π+ϵ/2, with ϵ≈0.2, then polygonal swimming behaviors in a plane orthogonal to the light direction are generated (11). While not investigated explicitly in our previous publication (11), we now show that this model also already accounts for negative phototaxis for π+ϵ/2<α<2π−ϵ/2, with ϵ≈0.2 (Fig. 3*A*, iii and Movie S4), consistent with previous experimental results (*SI Appendix*, Texts 3.3–3.5) (10).

For the light-dependent angular turn mechanism, we adapted the model to modulate the turning vector $K\hat{p}$ by making α dependent on light intensity. K depends on $\left|I\right|$ and signal $S=I\cdot \hat{r}\left(t\right)$ with corresponding coupling constants $K_{a}$ and $K_{d}$ for ambient and directional light components, respectively:

[1] $$\begin{array}{c} \begin{array}{cc} K(t) & =K_{0}+K_{a}\left|I\right|+K_{d}R\;\text{H}\left(S\right). \end{array} \end{array}$$

Here, $K_{0}$ is an intrinsic, light-independent reorientation rate and H is the Heaviside function accounting for eyespot shading. The response strength is given by R and is set to be equal to S here. The model exhibits positive phototaxis if α is confined to ϵ/2<α<π−ϵ/2 (Fig. 3*B*, iii and Movie S5), again with ϵ≈0.2.

For the photoresponse delay mechanism, we included a delay in signal transmission into the model, which is described by replacing the signal S with a delayed signal $S_{d}$:

[2] $$\begin{array}{c} \begin{array}{cc} S_{d}\left(t\right) & =0\;\text{if}\;t<t_{d}\\ S_{d}\left(t\right) & =S\left(t-t_{d}\right)\;\text{if}\;t\geq t_{d}, \end{array} \end{array}$$

where $t_{d}$ is the delay time of the response, and t=0 is the time when the light first reaches the photoreceptor. For delays on the order of half a roll cycle the model then exhibits positive phototaxis (Fig. 3*C*, iii and Movie S6).

For the photoresponse inversion mechanism, we introduced a phototaxis sign parameter P which captures this inversion of photoresponse due to light intensity increase vs. decrease over a threshold light intensity Δ:

[3] $$\begin{array}{c} \begin{array}{cc} S_{\mathrm{inv}}\left(t\right) & =PS(t)\\ P & =sgn(\left|I\right|-\Delta ). \end{array} \end{array}$$

The cell inverts its photoresponse via a parameter P, where P=−1 for positive phototaxis and P=1 for negative phototaxis. The cell reorients and exhibit positive phototaxis whenever the eyespot shades its photoreceptor and the cell detects darkness (i.e., $I\cdot \hat{r}<0$, Fig. 3*D*, iii and Movie S7).

Comparing the phase relation between K and $I\cdot \hat{r}$ (for all three mechanisms blue curve in Fig. 3*B*–*D*, iv), we find that for the first mechanism negative and positive phototaxis have the same phase relation (Fig. 3*A*, iv and *B*, iv, while the other two mechanisms show the opposite phase relation (Fig. 3*C*, iv and *D*, iv). Importantly, for all three mechanisms, the turning response $\left(K-K_{0}\right)sgn\left(\alpha -\pi \right)$ is opposite to detected light $I\cdot \hat{r}$ (red and blue curve in Fig. 3*B*–*D*, iv, respectively) in order to generate positive phototaxis (see *SI Appendix*, Text 3.4 for definition of turning response), while they are both aligned in order to generate negative phototaxis (Fig. 3*A*, iv). In the following, we will experimentally test which of the three mechanisms is actually realized in *E. gracilis*.

### Experimental Tests of the Three Mechanisms.

**Fig. 4.**
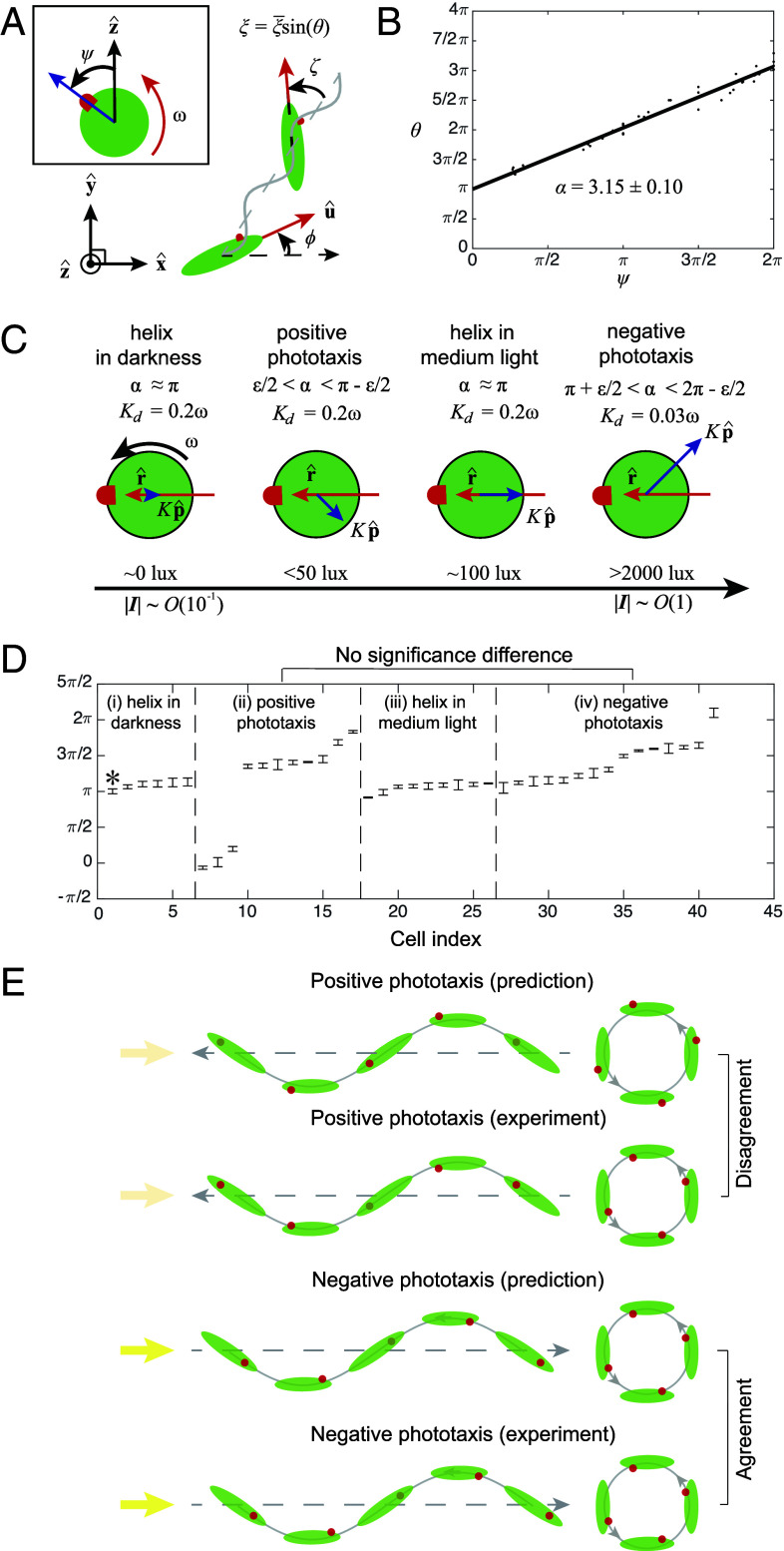
Experiments with freely swimming cells reveal that the light-dependent angular turn mechanism is not valid for *E. gracilis*. (*A*–*D*) Tracking of freely swimming cells shows that positive and negative phototaxis display a similar range of paw angle α. (*A*) Schematic defining ψ and θ. (*B*) Linear fit for the phase relation between ψ and θ gives α in i) darkness, ii) positive phototaxis, iii) medium light, and iv) negative phototaxis. (*C*) A schematic depicts the tuning of α and $K\hat{p}$ for different behaviors under the light-dependent angular turn mechanism. (*D*) α for different behaviors obtained from tracking of N=41 cells for at least three rolling cycles each. The error bars denote the SEM.The asterisk in (*D*) denotes the set of data presented in (*B*). (*E*) Schematics of predicted and experimentally observed eye spot orientation, which shows agreement for negative phototaxis but not for positive phototaxis.

To experimentally test the light-dependent angular turn mechanism (Fig. 3*B*), we measured the paw angle α for freely swimming cells under various conditions (*SI Appendix*, Text 4.3), i.e., i) helical swimming in darkness (∼0 lx), ii) positive phototaxis under directional stimulus (⪅50 lx), iii) helical swimming under medium background (∼100 lx), iv) negative phototaxis under high stimulus (>5,000 lx, where transition into negative phototaxis occurs at ∼2,000 lx) (Fig. 4*A*–*D* and *SI Appendix*, Text 4.3). In the two control cases of helical swimming under darkness (i) and medium background light (iii), α lies around π ($\overset{¯}{\alpha }=3.42\pm 0.06$ and $\overset{¯}{\alpha }=3.32\pm 0.07$), respectively, which agrees with the model predictions as α=π gives no bias toward or away from light. For negative phototaxis (iv), we obtained $\overset{¯}{\alpha }=4.40\;\pm \;0.23$, consistent with the expected range of π+ϵ/2<α<2π−ϵ/2. However, for positive phototaxis (ii), we obtained ($\overset{¯}{\alpha }=5.14\;\pm \;0.29$), which is not significantly different from α measured from negative phototaxis (P<0.0001), and does not agree with the expected range of ϵ/2<α<π−ϵ/2 (P<0.0001). This rules out the light-dependent angular turn mechanism (Fig. 4*E*).

**Fig. 5.**
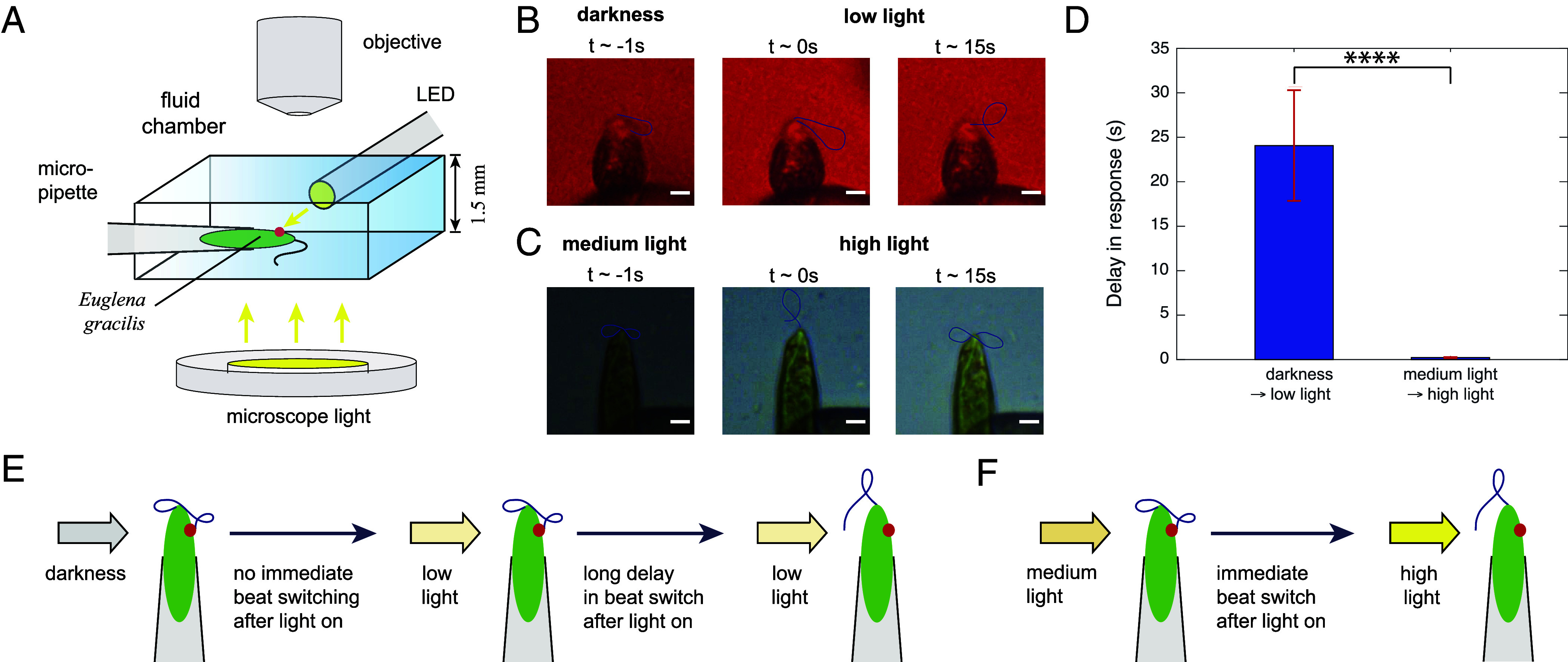
Experiments with spatially fixed cells reveal that the photoresponse delay mechanism is not valid for *E. gracilis*. (*A*) Schematic of the experimental setup for a *E. gracilis* held on a micropipette. (*B*) Procedure for measuring time delay for switching the beat state from darkness to low light intensities. (*C*) Procedure for measuring time delay for switching the beat state from medium light to high light intensities. (*D*) Delay in response during light step-up. The errorbars represent the SEM for the n=15 samples from N=6 cells. The large delay in response for the case of switching from darkness to low light indicates that the cell does not respond to the light step-up. The response is simply due to stochastic switching of beat pattern that appears even for the cell under darkness over times. (*E*) Schematics of the switching in beat state and the corresponding time delay observed from darkness to low light. (*F*) Schematics of the switching in beat state and the corresponding time delay observed from medium light to high light. (Scale bars, *B* and *C*: 5 μm.).

To experimentally test the delay mechanism (Fig. 3*C*), we used micropipette aspiration (22) to fix *E. gracilis* cells in place (Fig. 5*A*) and measure the response of flagellar beat pattern upon changes in light intensity. To mimic conditions of positive and negative phototaxis, the same background and stimulus light conditions (i–iv) as in Fig. 4*D* were used, except for (iv) the stimulus was >5,000 lx [to make sure no cells falling into transitional polygonal behavior at intermediate light intensities (11)]. Negative or positive phototaxis is triggered by tuning the microscope light intensity directly or applying an LED stimulus under a red observing light, respectively. We measured the time delay between the light stimulus turning on and the first beating state switching afterward (Fig. 5*D*). We found for the negative phototaxis condition, a delay of $t_{d}=0.23\;\pm \;0.06\;\;\text{s}$, while for the positive phototaxis condition, the delay of $t_{d}=24.06\;\pm \;6.21$s was significantly larger (n=15, P<0.0001). We also observed a very wide distribution of delays ranging from a minimum of 0.24 s to a maximum of 55 s for the positive phototaxis condition. We note that this wide distribution was not due to active responses of cells to light. This response time indicates the physiological stochastic switching in beat patterns so that cells turn toward a new direction over time. Importantly, we did not find a consistent delay difference between the positive and negative phototaxis condition on the time scale of half a roll cycle, i.e., ∼0.5 s, as would be required for inverting the swimming direction according to the delay mechanism (Fig. 3*C*). These results rule out the delay in photoresponse mechanism (Fig. 5 *E* and *F*).

**Fig. 6.**
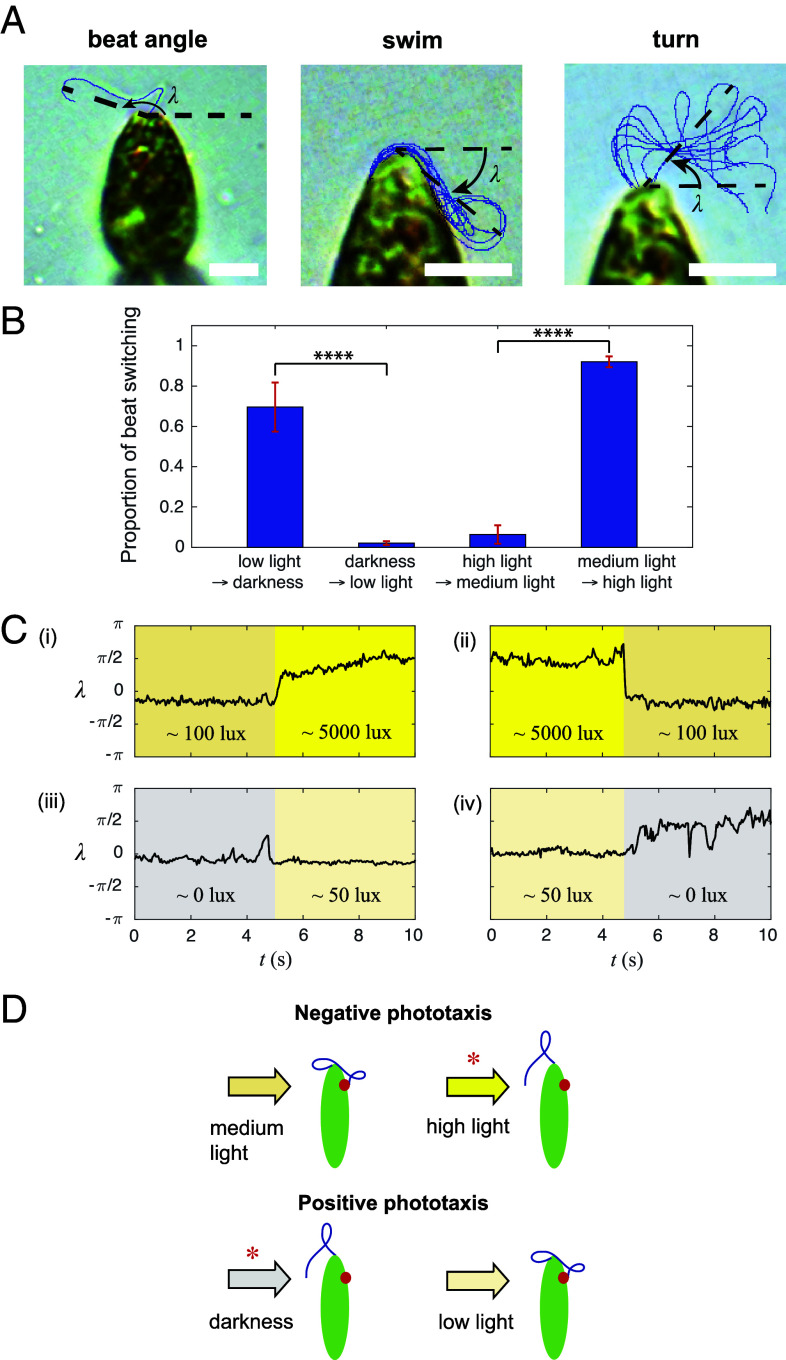
Experiments with spatially fixed cells reveal that the photoresponse inversion mechanism is consistent with *E. gracilis* light responses. (*A*) A beat angle λ is defined to quantify flagellar beats (*Left*). Two main beat patterns corresponding to swimming (*Middle*) and turning (*Right*) were observed, and that are equivalent to those observed previously [Fig. 1*D*, (11)]. (Scale bars, 5 μm.) (*B*) Proportion of beating state switching after light step-up and light step-down. The errorbars represent the SEM for the n=15 samples from N=5 cells. (*C*) Measurement of λ before and after light step-up and light step-down between different light intensities: 0 lx (gray), 50 lx (blue), 100 lx (pale yellow), 5,000 lx (yellow); typical traces shown. (*D*) *E. gracilis* coordinates between two beat patterns at different light intensities to achieve positive and negative phototaxis strategies, i.e., *E. gracilis* utilizes the “photoresponse inversion mechanism” (Fig. 3*D*); the red asterisks indicate an “activated turning state” due to changes in light intensity.

To experimentally test the photoresponse inversion mechanism (Fig. 3*D*), the observed beat patterns were quantified with the angle λ between the cell tip and the farthest material point on the flagellum within a beat cycle (Fig. 6*A*). We first measured the proportion of the beating state switching of cells held in place upon changes in light intensity (Fig. 6*B*), which captures the proportion of turning beat patterns within 5 s after light changes. For the negative phototaxis condition, we found that the cells have a significantly higher proportion of beat switching due to a step-up in light intensity (n=15, P<0.0001), while for positive phototaxis condition, the cells have a significantly higher proportion of beat switching due to a step-down in light intensity (P<0.0001). We then investigated the transient and adaptation behavior by periodically turning the stimulus on and off, with the on-period and the off-period lasting at least half a minute (*SI Appendix*, Text 4.4). In all cases, the cells displayed the two main beat patterns corresponding to helical swimming and turning behavior (Figs. 1*D* and 6*A*) as also established previously (11). Other intermediate beats would have a value of λ that is in between the one for the two main beat patterns. Under negative phototaxis light condition, the flagellum exhibits a swimming beat pattern under medium background light, while switching to the turning beat patterns almost immediately after exposure to a high stimulus light (Fig. 6*C*, i and Movie S8). When turning the stimulus light off, the beat state switched back to the swimming state (Fig. 6*C*, ii and Movie S9). Under the positive phototaxis condition, the flagellum stays at a swimming state when the LED is turned on, but once the LED was turned off, the flagellum switched between the two beat patterns stochastically (Fig. 6*C*, iii and iv and Movies S10 and S11). Hence the responses to changes in light intensity are fundamentally inverted between the negative and positive phototaxis conditions, i.e., the response is most robust and predictable as well as most immediate when the light intensity is increased vs. decreased, respectively (Fig. 6*B*). Furthermore, the cell responds with the turning vs. swimming beating pattern, respectively, once it perceives an increased light stimulus (Fig. 6*C*, i and iii). This implies a two-state feedback dynamics that applies to both positive and negative phototaxis under very high and very low light conditions, respectively (Fig. 6*D*). We note that while free-swimming cells may detect much more complex time-dependent light stimuli than those applied here, such complex stimuli are still related to these basic step-up/step-down stimuli and thereby likely evoke related flagellar responses as observed in these pipette experiments. We also observed stochastic switching and adaptation of beat patterns when the light intensity was held constant over longer times after an initial change in light intensity (*SI Appendix*, Text 4.4). The beat pattern then eventually recovers from the stochastic switching state to the normal swimming state after ∼100 s. All experimental results together are consistent with the prediction of the photoresponse inversion mechanism (Fig. 3*D*).

### Theoretical Performance of the Three Mechanisms.

**Fig. 7.**
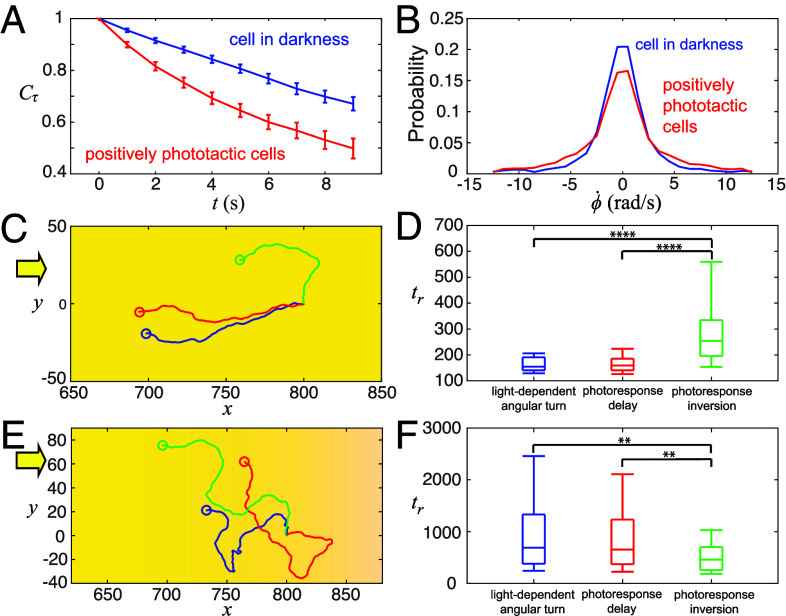
Depending on environmental and stimulus conditions, all mechanisms are effective, yet the photoresponse inversion mechanism has superior navigational performance when the cell has to overcome the sensory noise from its photoreceptor for positive and negative phototaxis. (*A*) Experimental measurements demonstrate that *E. gracilis* cells exhibiting positive phototaxis display a smaller displacement direction correlation $C_{\tau }$ over times (*SI Appendix*, Text 4.2) and (*B*) have a wider distribution of cell reorientation rate compared to cells in darkness. (*C*) Under parallel light rays, cells following the light-dependent angular turn (blue color) and photoresponse delay (red color) reach a light source faster than a cell following the photoresponse inversion mechanism (green color). (*D*) The box plots of $t_{r}$ for the three mechanisms for the case of parallel light rays shown in (*C*). (*E*) Under a weak light gradient, simulations show that a cell following the photoresponse inversion mechanism (green color) typically outperforms the cells following the light-dependent angular turn (blue color) and photoresponse delay (red color) to reach a light source. (*F*) The box plots of $t_{r}$ for the three mechanisms for the case of a weak light gradients as shown in (*E*). In (*D*) and (*F*), the midlines represent the median, and the box’s upper and lower bounds indicate the interquartile range; the upper and lower whiskers denote the 9th and 91st percentile of the simulation data, respectively. 100 simulations were performed for each mechanism.

This raises the question of why the inversion mechanism is used in *E. gracilis*, and we hypothesize that it provides a significant performance advantage under relevant conditions (and potentially it might also be easier to implement on a molecular or cellular level, and it might be easier to evolve). To test that, we quantified the experimentally measured mean free path and reorientation rate of the biased random walk of *E. gracilis* during positive phototaxis: compared to the cells in darkness, the positively phototactic cells have a shorter mean free path, indicated by the rapid decrease in correlation of displacement direction (i.e., smaller $C_{\tau }$ over times, n=50, P<0.01, Fig. 7*A* and *SI Appendix*, Text 4.2). Moreover, the positively phototactic cells reorient more frequently, as indicated by the wider distribution of the reorientation rate with a significantly larger variance (i.e., σ=10.3$\;{\text{rad}}^{2}/\;{\text{s}}^{2}$ for cells under darkness; σ=15.7$\;{\text{rad}}^{2}/\;{\text{s}}^{2}$ for cells under positive phototaxis; f-test, n=500, P<0.0001, Fig. 7*B* and *SI Appendix*, Text 4.2). Hence noise seems to play a significant role during positive phototaxis.

We then considered the typical noise types and levels involved in positive phototaxis and the resulting effects on the swimming paths and ultimately on taxis behavior. The light intensity during positive phototaxis in our experiments (Fig. 2 *C* and *D*) is ∼50 lx and thereby potentially reflects natural settings, and which corresponds to $2\times 10^{6}$ photons/s hitting the photoreceptor (*SI Appendix*, Text 3.7). There is evidence that flavoproteins and flavins are responsible for *E. gracilis*’s photoreception (10), which feature a low absorption rate of photons (∼1%) (54), thus the effective light signal would be $2\times 10^{4}$ photons/s. Moreover, the thermal noise for a flavin-based photoreceptor is around $10^{4}$ photons/s (55), which is comparable to the effectively detected light signal. In contrast, the light signal for negative phototaxis [>2,000 lx (8, 28, 29)] is typically 80-fold larger compared to the sensory noise, which is then likely sufficient for the cell to discriminate the signal direction from noise and therefore to exhibit a directional response (Fig. 2 *A* and *B*). We also estimate that the Brownian noise does not play a significant role given the comparably large cell body size of *E. gracilis*, e.g., the rotational diffusion due to thermal noise is $D_{r}∼1.5\times 10^{-6}$ rad^2^/s (*SI Appendix*, Text 3.2), and even the rotational diffusion due to active reorientation of the cells in the dark was measured to be only 0.02 rad^2^/s (*SI Appendix*, Text 3.2) while *E. gracilis* can make course corrections of about 0.35 rad within a single beat stroke, i.e., within 50 ms (see below). Hence, we propose that the main noise limitation for *E. gracilis* cells during positive phototaxis at low light conditions stems from receptor noise, while for negative phototaxis at high light conditions, noise does not appear to play any role; hence, any search mechanism needs to particularly overcome these noise effects at low light conditions.

Based on these considerations, we incorporated translational and rotational diffusion as well as sensory noise into all three mechanisms and then simulated their phototaxis performances under low light levels and low signal-to-noise levels (*SI Appendix*, Texts 3.5 and 3.6):

[4] $$\begin{array}{c} \begin{array}{c} S\left(t\right)=I\cdot \hat{r}\left(t\right)+I_{\epsilon }n\left(t\right). \end{array} \end{array}$$

Here, S(t) is the perturbed signal detected by the cell, $I_{\epsilon }$ is the noise strength, and n(t) is a random vector with components following a Gaussian distribution with zero mean and unit variance. Based on the experiments shown in Fig. 6*C* and *SI Appendix*, Text 4.4, we now need to also account for the stochastic beat switching in the case of the photoresponse inversion model, hence we now use for R in Eq. **1**:

[5] $$\begin{array}{c} \begin{array}{c} R(t)=\xi m(t). \end{array} \end{array}$$

Here, ξ sets the maximum response and m(t) is a random variable with a uniform distribution in [0,1]. We then determined the theoretical performance of the different mechanisms by measuring the time $t_{r}$ required for a cell to travel a specific distance of 100 cell body lengths toward the light source. We found that in the absence of attenuation of light, i.e., the light intensity $\left|I\right|$ being constant everywhere, cells following the photoresponse inversion mechanism typically reach the goal slower than cells using the other two mechanisms (n=100, P<0.0001, Fig. 7 *C* and *D* and Movie S12). Conversely assuming that light intensity attenuates over ecologically relevant values (and as mimicked in our experimental conditions) (*SI Appendix*, Text 3.6), we found the photoresponse inversion mechanism performs significantly better than the other two (n=100, P<0.01, Fig. 7 *E* and *F* and Movie S13): the amount of light at a location x is given by combining Lambert’s law and inverse square law (56):

[6] $$\begin{array}{c} \begin{array}{c} \left|I\right|=I_{0}c\frac{e^{-\beta \left|x-x_{0}\right|}}{{\left|x-x_{0}\right|}^{\gamma }}. \end{array} \end{array}$$

Here, $I_{0}$ is the light intensity at a reference location $x_{0}$. c, β, and γ are coefficients accounting for optical aberration, absorption, and path loss, respectively. For typical ecological conditions as well as for our experimental conditions, the parameters are c=0.74, β=0.29, and γ=0.056; hence, light intensity approximately decreases by 1/4 to 1/2 when doubling the distance (*SI Appendix*, Text 3.6).

From visually inspecting the simulated swimming trajectories, it appears that under a weak light condition, the photoresponse inversion mechanism allows a cell currently swimming in a wrong direction to correct its swimming direction by a stochastic response, while the other two mechanisms heavily depend on detecting a sufficiently high light intensity and thereby fail to correct the swimming direction if the signal is too weak. These results suggest that the photoresponse inversion is a more robust mechanism under weak and noisy light conditions and an intrinsically noisy sensor.

## Discussion

**Fig. 8.**
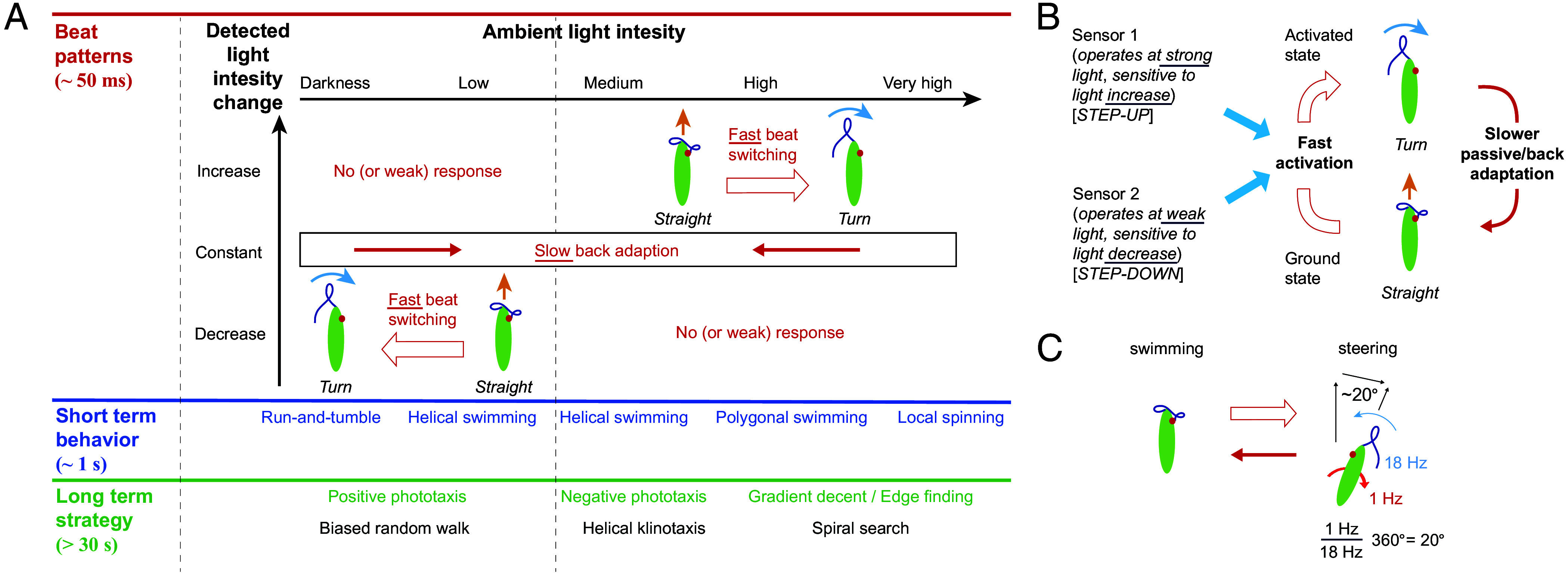
*E. gracilis* utilizes the photoresponse inversion mechanism to overcome the sensory noise from its photoreceptor for positive and negative phototaxis. (*A*) Proposed behavioral phase space based on results of this paper and the much wider previous literature. Switching between the two beating patterns at short time scales leads to various medium term photobehaviors and various long term phototaxis strategies. (*B*) “Two sensor/two-beat state (inversion) model”: two photosensors actuate the “turning beating state,” where one sensor actuates based on light decrease at very low intensity, and the other sensor actuates on light increase at high light intensity. The switching back to the “forward swimming beating state” likely happens passively on slower times scale by adapting to the changed light intensity over time (here we hypothesize that the backswitching cannot be directly actuated, but a reverting back to the original light intensity might speed up the adaptation process). (*C*) Illustration on how discreteness of the beat as well as the amount of angular change per beat cycle relative to the number of beats for one full roll ultimately determines the orientational steering resolution.

In summary, we proposed multiple theoretical mechanism of how microswimmers in principle could select between various phototaxis strategies based on light conditions (Fig. 3) (11), and we conclude that for *E. gracilis* the “photoresponse inversion” is the only suitable mechanism consistent with all data presented here (Fig. 8*A*) and in the literature (9, 10, 29), while we ruled out the other two mechanisms for this organism. This highlights an interesting solution for light searching based on a two-state feedback dynamics that applies to both positive and negative phototaxis under very high and very low light conditions, respectively (Fig. 6*D*): the cell mainly swims with helical beats in its favorable light conditions (low or medium background light), and switches to turning beat patterns for reorientation when it experiences unfavorable light conditions (darkness or high light). This simple feedback mechanism then leads to “helical klinotaxis” (a directional steering mechanism by tuning the paw angle α) in the case of high signal-to-noise levels during negative phototaxis, and to a “biased random walk” (run-and-tumble strategy) in the case of low signal-to-noise levels during positive phototaxis (14, 52), enabling effective phototaxis strategies over a very large range of light intensities (i.e., from 0 lx to over 10,000 lx).

Importantly, the underlying flagella responses at low vs. at high light intensities are inverted (Fig. 8 *A* and *B*), i.e., at low intensity, flagella beating primarily reacts to a decrease in light intensity (“step-down”) vs. at high intensity to an increase in light intensity (“step-up”), in line with prior research (10, 11, 27, 44). In both cases, the “step-response” represents a fast actuation from a “forward swimming beat state” (“ground state”) to a “turning beat state” (“activated state”), and with a backswitching upon stimulus reversal or with a much slower (likely passive) backrelaxation in case the stimulus stays constant (Fig. 8*B*). Previously, the actuation and backrelaxation time scales for both step-responses were proposed to be different as likely different photoreceptors are involved (57, 58). We now determined these actuation and backrelaxation time scales for the step-up responses to be 0.23 ± 0.06 s (consistent with ref. 39) and 25 ± 7 s, respectively, and for the step-down responses to be 1.0 ± 0.3 s and 24 ± 6 s, respectively. Hence the actuation is 1–2 orders of magnitude faster than the relaxation, furthermore, the actuation for the step-up response is significantly faster than for the step-down response, while the relaxation time scales appear to be equal for both responses.

Our experimental and modeling results then posit that the coordinated and sequenced switching between just two beat states caused by the momentarily sensed light intensity (also accounting for sensor adaptation) provides a concise explanation for the large set of versatile and complex photobehaviors exhibited by *E. gracilis* (Fig. 8*A*), e.g., positive and negative phototaxis along the light direction, polygonal search perpendicular to the light vector in complex light fields, transitioning between normal vs. anomalous diffusion, and localized spinning (8–11, 27, 29, 30, 35, 36, 44). We note that additional photobehaviors have been reported that potentially require more complex explanations, e.g., photokinesis [i.e., the change in velocity based on light intensity changes (10)], swimming at an angle relative to the vector of polarized light (43, 59), or the swimming direction when subjected to multiple light sources from different directions (48). Our model can already recapitulate some of these aspects (*SI Appendix*, Text 3.8); these behaviors deserve further experimental and theoretical study but which is outside of the scope of this paper and left for future work. Overall, our work now synthesizes many earlier findings and resolves open questions regarding the relationship between step responses, flagellar beat patterns, and cell steering (Figs. 5*B* and 6*A*)—work that historically had to mainly rely on whole cell behavioral observations and with much less detail regarding the flagellar beat patterns being available given the lack of digital high-speed imaging technology at the time (10, 44).

These results then also quantify the limits of taxis performance and steering precision (Figs. 2 *B* and *C* and 7*B*). Generally, *E. gracilis* operates with discrete beat strokes leading to discrete (“quantized”) steering, the cellular motion is subjected to translational and rotational Brownian motion, the photosensors and molecular signaling cascades are subjected to noise including photon-shot-noise, and the light stimulus might be noisy. For high light intensity (negative phototaxis), the limit for path correction should be ∼20^°^ per beat, as it takes a cell ∼1 s to roll around its long axis (11), during which the cell either executes a single correcting turning beat or none at all (of course, in the case of larger course corrections, the cell can also execute multiple beats per roll). We measured a course change of ∼20^°^ per beat previously (11) (Fig. 8*C*). Related arguments were also summarized before but not connected quantitatively to the single beat (10). This also aligns with our measured stdev of reorientation rates of ∼70^°^/s (*SI Appendix*, Fig. S8*B*), which implies that cells with helical pitch have reorientation angles of ∼20^°^ (*SI Appendix*, Text 4.2). For low light conditions (positive phototaxis), we already emphasized that the thermal noise for a flavin-based photoreceptor amounts to a sensory noise of ∼10^4^ photons/s (55), which is comparable to the effectively detected light signal (see above). For perfect steering, we would again expect ∼20^°^/s (Fig. 8*C*), but given the sensor noise, a significant proportion of wrongly missed or added steering actions are expected, thereby limiting this resolution further. By comparing the experimentally measured reorientation rates of cells in darkness (1 SD = 1.88±0.14 rad/s, 95% CI, Fig. 6*B*) with that of cells under positive phototaxis (1 SD = 2.57±0.29 rad/s, 95% CI, Fig. 6*B*), we can estimate the steering resolution to be ∼40^°^/s, i.e., corroborating our expectation that this steering resolution for positive phototaxis is lower than the one for negative phototaxis. Note also that for these estimated performance limits the corrective light signal changes and consequently activation energy should be sufficiently low so that the backswitching time is short, i.e., within a single flagellar beat. These different signal-to-noise levels then also explain the chosen taxis strategies, i.e., at high light intensities the cell can take the fastest, nearly straight swimming path via the helical “klinotaxis” strategy, while at low light intensity, the system overall operates at its noise limit and therefore has to sample for longer via the less direct “biased random walk” strategy.

How the selection of these different photobehaviors at different light intensity scenarios is achieved at a subcellular and molecular signaling cascade level is not fully understood (41, 42, 57, 58, 60) and beyond this work. But in short, there is strong evidence that positive and negative taxis are regulated by different flavin-based photosensors, e.g., a family dissimilar PACs, and that they then likely feed into the same flagella activation mechanism (41). Having two different photosensors might be advantageous for at least two reasons: 1) being optimally responsive to a decrease vs. an increase in light intensity (i.e., step-down vs. step-up, respectively), and 2) being optimally sensitive to very low vs. very high light intensities.

These biophysical constraints on phototaxis may also explain the relative and absolute placement of eyespot and flagellum, and why *E. gracilis* is a puller-type swimmer (17). Intuitively, it is advantageous for the flagellum to be close to the photoreceptor to aid a fast and robust signaling. Furthermore, during negative phototaxis under highest light intensity, the photosensory organ is then more protected against photodamage (and is also more sensitive) by being shaded from the whole cell body, while during positive phototaxis, the sensor points mostly toward the light source and is thereby able to capture more of the scarce photons that otherwise would be adsorbed by the cell body. This design rule might be extensible to other phototactic cells such as *C. reinhardtii* (20, 22).

The three proposed phototaxis mechanisms (Fig. 3) may inform the design of other natural and even synthetic microswimmers, with implications for ecology, sustainability, and biotechnology. Microorganisms (e.g., *C. reinhardtii* and *Gonium pectorale*) or small animal larvae (e.g., *Platynereis dumerilii*) swim in helical paths during phototaxis to enhance their light sensing (18, 20, 61–63). And although we ruled out the light-dependent angular turn mechanism for positive phototaxis of *E. gracilis*, a recent study indicates that the phase relationship captured by this mechanism agrees with both positive and negative phototaxis for *C. reinhardtii* (64), and where the eyespot had been reported to be located correspondingly at the outer vs. inner side of the helix during positive vs. negative phototaxis, respectively (19, 63). We also expect that the delay in photoresponse mechanism may have implications for the selection of the phototaxis sign in other phototactic organisms (61). A deeper biophysical comparison between these (and other) organisms would be valuable for future work. Synthetic microswimmers (65–68) that can select between positive and negative taxis in response to environmental stimuli (e.g., chemicals, light, flows) could be designed with and controlled by such simple noisy sensor and two-state feedback responses. This will aid the understanding and engineering of natural or genetically modified microswimmers, especially for sustainability application, such as waster water treatment, bioremediation, and bioreactors for chemical production (1–5).

## Materials and Methods

All experimental materials and methods are detailed in *SI Appendix*, Text 2; all theoretical and simulation methods are detailed in *SI Appendix*, Text 3.

In short: *E. gracilis* was obtained from Carolina Supplies (#152800). The cell culture could be readily used for negative phototaxis experiments. For positive phototaxis experiments, the cells were kept in the dark for 24 h before the experiments such that the cells were fully adapted to darkness before any light stimuli were introduced. Cells were imaged using bright field microscopy on a Leica DM500 compound microscope. LEDs were used and voltage tuned to provide light stimuli of different intensities to elicit positive and negative phototaxis. In some experiments, cells were held in place via micropipette aspiration. Cells and flagella were digitally imaged with frame rates between 10 and 400 fps. Image data were analyzed with computational support or manually (see details in *SI Appendix*, Texts 4.2–4.4). Modeling and numerical integration was performed in Matlab.

## Supplementary Material

Appendix 01 (PDF)

Movie S1. Negative phototaxis experiment. The video is replayed at the real time.

Movie S2. Positive phototaxis experiment. The video is replayed at the real time.

Movie S3. Negative phototaxis experiment, individual cell shows beat switching. The images were sampled at 200 fps and the video is replayed at 10× slower than the real time.

Movie S4. Negative phototaxis simulation.

Movie S5. Positive phototaxis simulation, light-dependent angular turn.

Movie S6. Positive phototaxis simulation, delay in photoresponse.

Movie S7. Positive phototaxis simulation, inversion in photoresponse.

Movie S8. Micropipette experiment, medium light to high light. The images were sampled at 200 fps and the video is replayed at 10× slower than real time.

Movie S9. Micropipette experiment, high light to medium light. The images were sampled at 200 fps and the video is replayed at 10× slower than real time.

Movie S10. Micropipette experiment, dark to low light. The images were sampled at 200 fps and the video is replayed at 10× slower than real time.

Movie S11. Micropipette experiment, low light to dark. The images were sampled at 200 fps and the video is replayed at 10× slower than real time.

Movie S12. Simulations of the three mechanisms, parallel light.

Movie S13. Simulations of the three mechanisms, light gradient.

## Data Availability

Raw movies and object-tracking data from movies have been deposited in GitHub (https://github.com/alancht/Euglena) (69). All other data are included in the manuscript and/or supporting information.
